# Influence of TiO_2_, Al_2_O_3_, and Basicity on Viscosity and Structure of High Titanium-Bearing Blast Furnace Slag

**DOI:** 10.3390/ma16072575

**Published:** 2023-03-24

**Authors:** Wenbo Zhou, Tingle Li, Dong Lan, Changyu Sun, Songtao Yang

**Affiliations:** 1 School of Materials and Metallurgy, University of Science and Technology Liaoning, Anshan 114051, China; 2 Fujian Sansteel (Group) Co., Ltd., Sanming 365000, China

**Keywords:** blast furnace slag, slag viscosity, slag structure, TiO_2_

## Abstract

The viscosity of high-titanium blast furnace slag with different TiO_2_ content, Al_2_O_3_ content, and basicity was measured at 1653–1773 K using the rotational cylinder method. The phase composition of the slag is measured by XRD. Phase diagram of the slags is calculated by FactSage software. Ionic network structure of the slags is analyzed by FT–IR. Results show that TiO_2_ depolymerizes the silicate network structure, reducing viscosity at high temperature, while increasing Al_2_O_3_ content generates a more complicated silicate, increasing viscosity. Basicity affects viscosity, with higher basicity resulting in lower viscosity above 1733 K. Perovskite significantly affects the viscosity of slag. This study provides an in-depth understanding of the relationship between the composition and viscosity of high-titanium blast furnace slag, which is very important for improving production efficiency.

## 1. Introduction

Viscosity is an important physical property of slag to determine the stability and productivity of the blast furnace iron-making. Conventional blast furnace slag mainly contains CaO, SiO_2_, MgO, and Al_2_O_3_. When vanadium–titanium ore is used as a raw material for blast furnace iron-making, the softening and melting zone of the furnace changed [1,2,3,4,5,6]. Most of the TiO_2_ enters the blast furnace slag, making it sticky and causing difficulties in separating slag and iron in the furnace hearth. The viscosity and structure of the blast furnace slag changed due to the addition of TiO_2_ [7,8,9]. 

High titanium-bearing blast furnace slag is the main type of blast furnace slag produced by vanadium and titanium ore in the Panzhihua area during blast furnace smelting, which is mainly composed of oxides such as CaO, SiO_2_, MgO, Al_2_O_3_, and TiO_2_, and when the temperature is constant, the viscosity of the slag mainly depends on these oxide components [10,11]. Some investigations on the viscosity of titanium-bearing slag have been carried out. It is generally believed that the viscosity decreases with the increase in TiO_2_ content [12,13]. However, there are still different opinions about the mechanism of the influence of TiO_2_. One opinion is that TiO_2_ as the network former forms the simpler structure of TiO_4_^4−^ monomers. So, the strength of the network structure is weakened [11,14,15]. Another opinion is that TiO_2_ acting as a basic oxide in the slag depolymerizes the silicate network structure [16,17].

Suitable basicity plays a crucial role in the blast furnace smelting process. Excessive basicity leads to an increase in high-melting-point compounds in the slag, resulting in poor fluidity of blast furnace slag and difficulty in separating slag and iron. If the basicity is too low, the desulfurization capacity of blast furnace slag decreases, and at the same time, the blast furnace slag erodes the furnace lining. On the other hand, it is well-known that Al_2_O_3_ is a typical amphoteric oxide. In the CaO-MgO-SiO_2_-Al_2_O_3_ slag system, whether Al_2_O_3_ acts as an acidic oxide or as an alkaline oxide depends on the slag composition. In some slags, Al_2_O_3_ is more inclined to act as a network former, and the viscosity increases with Al_2_O_3_ addition [18,19]. So, the effect of Al_2_O_3_ on the viscosity of the slag with different composition is different. In other slags, the viscosity first increases and then decreases with Al_2_O_3_ addition [20,21]. In the smelting of vanadium–titanium ore in a blast furnace, the physical and chemical properties, as well as the composition of the liquid phase line temperature and viscosity of the blast furnace slag, constantly change during the smelting process. These parameters have a significant impact on the smooth flow of the smelting process. However, there are a few reports on the physical and chemical properties of high-titanium slag. Therefore, obtaining these physical and chemical parameters through systematic testing is of great practical significance for optimizing the smelting process, reducing energy consumption, and improving production efficiency.

This paper investigates the impact of TiO_2_, Al_2_O_3_, and basicity (R = m(CaO)/m(SiO_2_)) on the viscosity of CaO-SiO_2_-MgO-Al_2_O_3_-TiO_2_ slags. Fourier transform infrared (FT–IR) spectra of the water-quenched slag is used to study the relationship between the network structure and viscosity. X-ray diffraction (XRD) analysis is conducted on the water-quenched slag at various temperatures to determine the liquidus isotherm using Factsage 7.2. This information is used to examine the effect of solid precipitates in the slag on viscosity. The study aims to provide a theoretical basis for utilizing vanadium–titanium ore in blast furnace and analyze the impact of these variables on blast furnace slag viscosity. 

## 2. Materials and Methods

Preparing slag by mixing pure oxides (MgO, SiO_2_, Al_2_O_3_, TiO_2_) and pure CaCO_3_ as the source of CaO. First, 200 g of slag powder was pre-melted at 1773 K for 1 h, then the sample was removed and cooled to room temperature by water cooling to obtain a water-quenched slag as an experimental sample. About 20 g slag samples were used to carry out the qualitative FT–IR and XRD characterization of the slags and an additional slag sample was used to measure the viscosity. The calculated chemical composition of the slag is shown in Table 1. The design composition of the slag is based on the actual composition of the Pangao steel plant [8,9,10]. 

**Figure 1:**
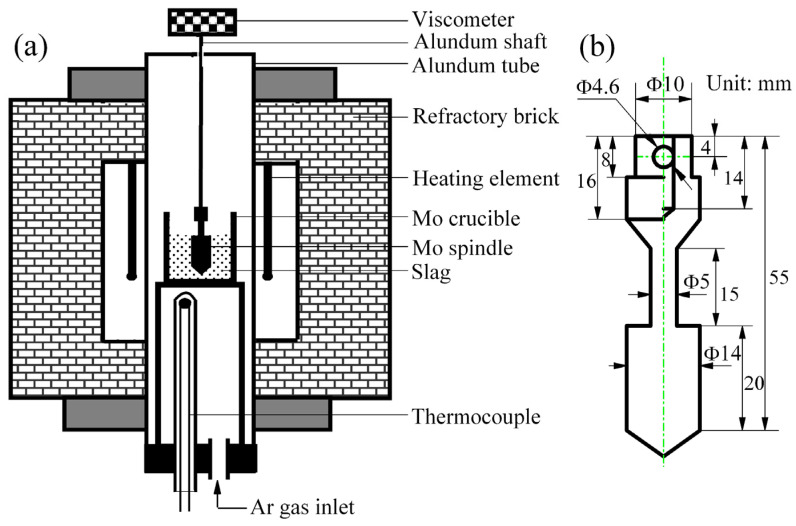
The schematic diagram of viscosity measurements. (**a**) Experiment apparatus; (**b**) Mo spindle.

In this study, the slag viscosity was measured using the rotating-cylinder method [22]. The experimental equipment used is a high temperature melt property tester (RT-3, Mingjian Hi-Tech Industrial Co., Suzhou, China). Figure 1a shows the experimental apparatus, which consists of an electric resistance furnace equipped with U-shaped MoSi_2_ heating elements for system heating. A Mo crucible with a height of 80 mm and an inner diameter of 40 mm was used to hold a 140 g sample, which was heated at a rate of 5 K/min up to 1773 K with a constant flow of Ar gas (500 mL/min). Viscosity measurement was conducted at every 40 K interval during cooling, with an equilibration time of 30 min at each temperature. The rotating spindle, as detailed in Figure 1b, was set to rotate at a speed of 300 rev/min, and five viscosity measurements were taken at the same temperature. The viscosity reported in this paper is an average of these five measurements. 

## 3. Results and Discussion

The viscosity of the CaO-SiO_2_-MgO-Al_2_O_3_-TiO_2_ slags with different basicity, TiO_2_ content, and Al_2_O_3_ content are shown in the Table 2. The viscosity increases with the decrease in temperature. When the temperature decreases to 1653 K, the viscosity shows a large increasing trend in some slag systems. 

### 3.1. Effect of TiO_2_ on Viscosity and Structure

**Figure 2:**
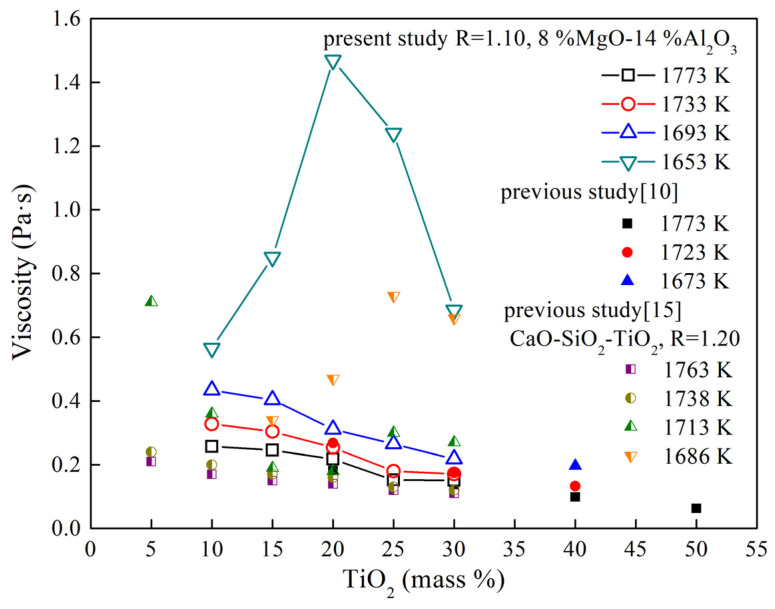
Viscosity of the CaO-SiO_2_-8 mass% MgO-14 mass% Al_2_O_3_-TiO_2_ (R = 1.10) slag as a function of mass% TiO_2_.

Figure 2 shows the effect of TiO_2_ content on viscosity of the CaO-SiO_2_-8 mass% MgO-14 mass% Al_2_O_3_-TiO_2_ slag with R = 1.10. The viscosity decreases with the increase in TiO_2_ content from 10 mass% to 30 mass% when the temperature is greater than 1693 K, which is consistent with previous study [11,15]. However, at 1653 K, the viscosity first increases and then decreases with the increase in TiO_2_ content, and it shows a maximum when TiO_2_ content is 20 mass%. This is different from the previous studies, where it was found that the viscosity of CaO-SiO_2_-TiO_2_ slag is the highest when the TiO_2_ content is 25% by mass [15].

**Figure 3:**
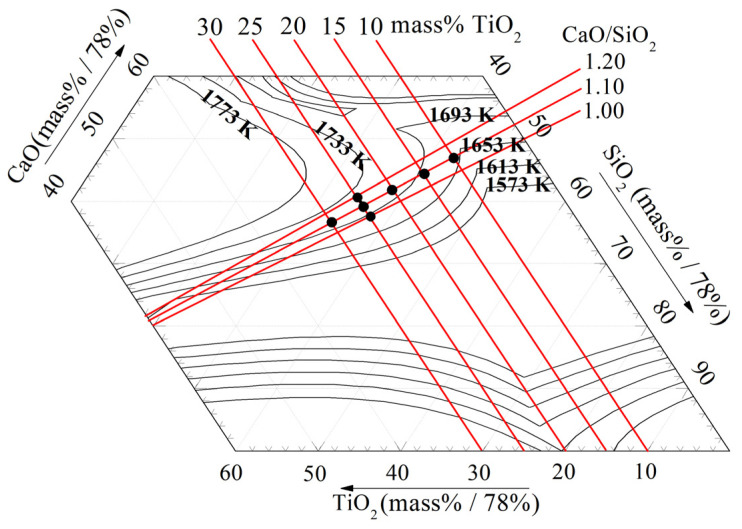
Phase diagram of CaO-SiO_2_-8 mass% MgO-14 mass % Al_2_O_3_-TiO_2_ slag, K.

Figure 3 shows the isotherm phase diagram calculated by FactSage [23]. At temperatures above 1693 K, the composition of the slag is entirely in liquid phase, and its viscosity is controlled by the network structure of the liquid phase. As the TiO_2_ content in the slag increases, the viscosity exhibits a decreasing trend. This can be attributed to the fact that the ionic radius of Ti^4+^ is about 1.5 times larger than that of Si^4+^, resulting in a weaker bond between Ti^4+^ and O^2−^ compared to that between Si^4+^ and O^2−^ [8]. Consequently, the formation of TiO_4_^4−^ monomers weakens the network structure [15]. On the other hand, the effect of Al_2_O_3_ content on viscosity is different from that of TiO_2_, despite the larger ionic radius of Al^3+^ compared to that of Si^4+^ in the slag [18,24]. Therefore, the reason for TiO_2_ addition leading to a decrease in viscosity is not yet clear, although it is possible that TiO_2_ may exist as the basic oxide and depolymerize the network structure in the present slag systems.

**Figure 4:**
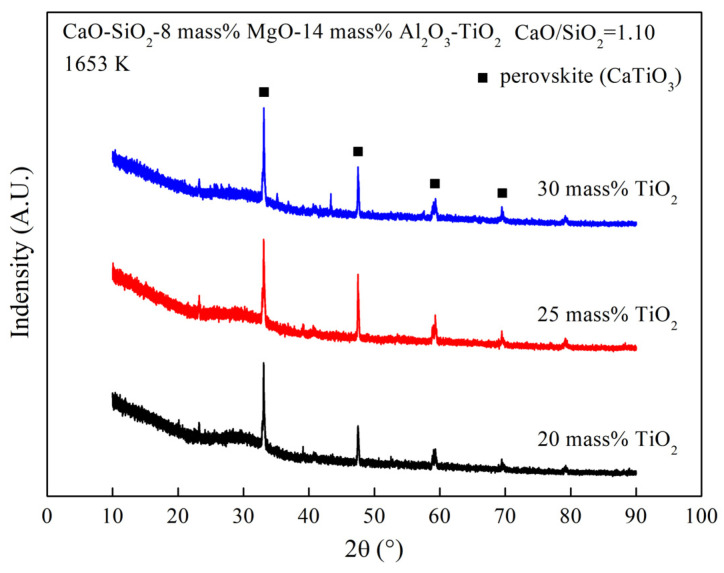
XRD curves of the slag containing different TiO_2_ content at 1653 K.

It can be seen from Figure 3 that liquidus temperature of the slags is greater than 1653 K. Perovskite is present in the slag at 1653 K, according to the XRD curves of the slags in Figure 4. So, the viscosity should be affected by the solid phase precipitated from the slag at low temperatures. As can be seen from Figure 3, the 1653 K isothermal curve is much further away from the composition points of slags containing 15 mass% to 25 mass% TiO_2_ than from slags containing 10 mass% and 30 mass%. It can be considered that the former have more solid phase in the slag than the later. The content of the solid phase plays a key role in the slag viscosity at 1653 K. Hence, the viscosity increases first and then decreases with TiO_2_ content from 10 mass% to 30 mass%.

### 3.2. Effect of Basicity on Viscosity and Structure

**Figure 5:**
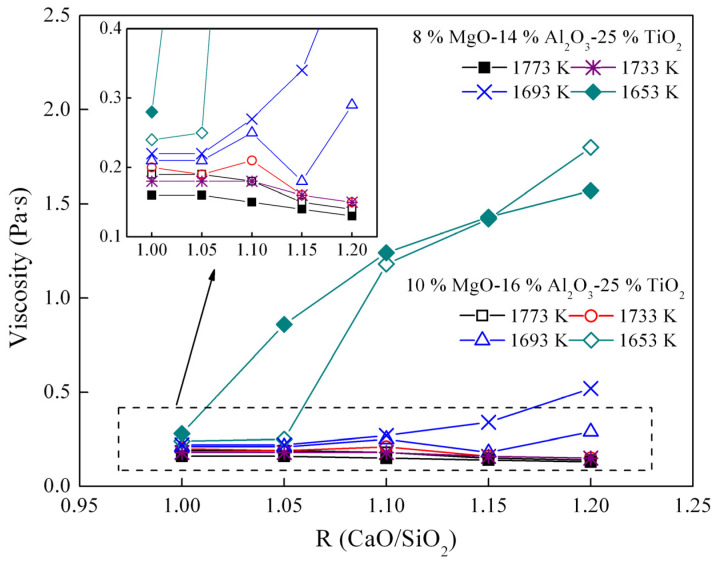
Viscosity of the CaO-SiO_2_-MgO-Al_2_O_3_-25 mass% TiO_2_ slag as a function of basicity.

Figure 5 illustrates how basicity affects the viscosity of the CaO-SiO_2_-Al_2_O_3_-MgO-25 mass% TiO_2_ slag. As shown, the viscosity of the slag decreases with increasing basicity when the temperature is greater than 1733 K. This can be attributed to the network structure, as indicated in Figure 3, where the liquidus temperature of the slag with a basicity ranging from 1.00 to 1.20 is lower than 1733 K. CaO serves as a network modifier, which depolymerizes the network structure, whereas SiO_2_ acts as a network former, which polymerizes the network structure. The rise in basicity corresponds to an increase in CaO content and a decrease in SiO_2_ content. In other words, an increase in basicity reduces both the size and number of the silicate network structure, leading to a decrease in viscosity.

**Figure 6:**
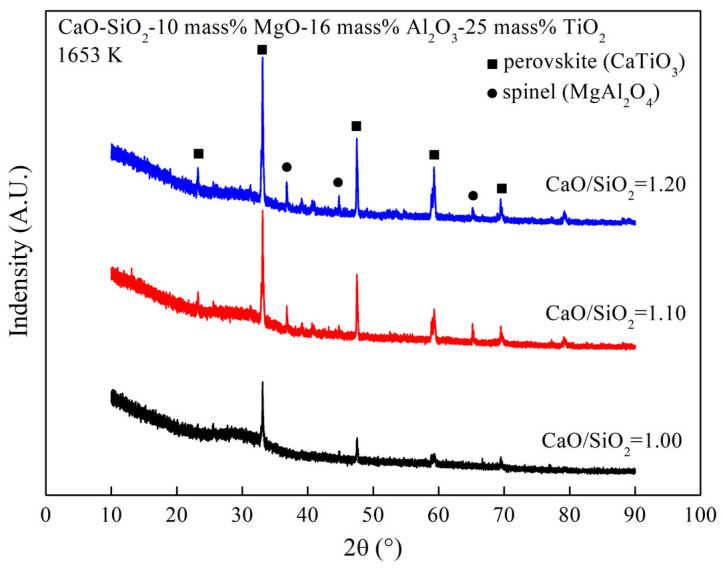
XRD curves of the slag containing different basicity at 1653 K.

Figure 6 shows the XRD results of the CaO-SiO_2_-16 mass% Al_2_O_3_-10 mass% MgO-25 mass% TiO_2_ slag system at different basicity levels at 1653 K. The solid phase of the slag contains calcium titanate and spinel phases. As the basicity increases from 1.00 to 1.20, the content of the solid phase in the slag increases significantly, leading to an increase in viscosity at 1653 K. In particular, although there are solid phase points with an basicity of 1.00 in the slag at 1653 K, the viscosity is still low. This may be because the solid phase is mainly dispersed in the liquid phase, and when the amount of solid phase is small, it does not form large aggregates. The formation of large aggregates by the solid phase has almost no effect on the viscosity.

### 3.3. Effect of Al_2_O_3_ on Viscosity and Structure

**Figure 7:**
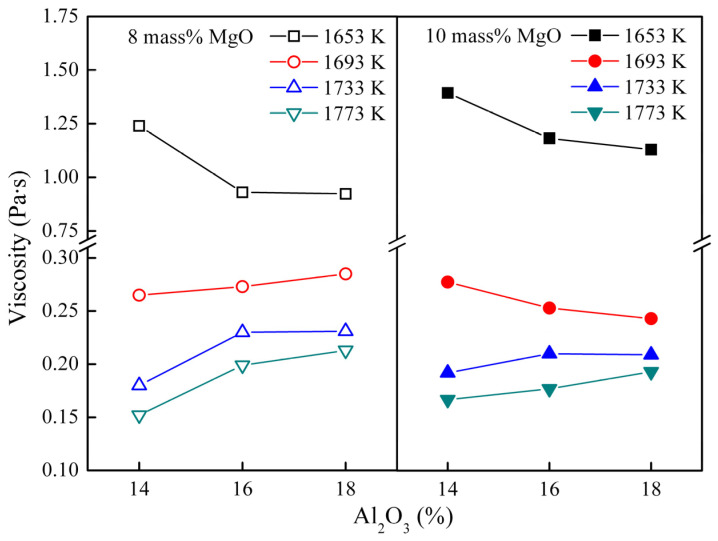
Viscosity of the CaO-SiO_2_-MgO-Al_2_O_3_-25 mass% TiO_2_ (R = 1.10) slag as a function of mass% Al_2_O_3_.

Figure 7 shows the effect of Al_2_O_3_ on viscosity of the CaO-SiO_2_-Al_2_O_3_-MgO-25 mass% TiO_2_ slag with R = 1.10. The viscosity increases with the increase of Al_2_O_3_ content in the slag at 1733 and 1773 K, and the viscosity decreases at 1653 K. At 1693 K when the Al_2_O_3_ content increases, the viscosity of the slag with 8 mass% MgO decreases and the viscosity of the slag with 10 mass% MgO increases, with the viscosity of the two contents changing in opposite patterns. It is widely believed that Al_2_O_3_ acts as an amphoteric oxide in the slag [25,26,27,28]. The amphoteric oxide may show different properties in different slag system [21,26]. In the present study, Al_2_O_3_ may be expected, as a network former, to cause an increase in the viscosity at high temperature.

**Figure 8:**
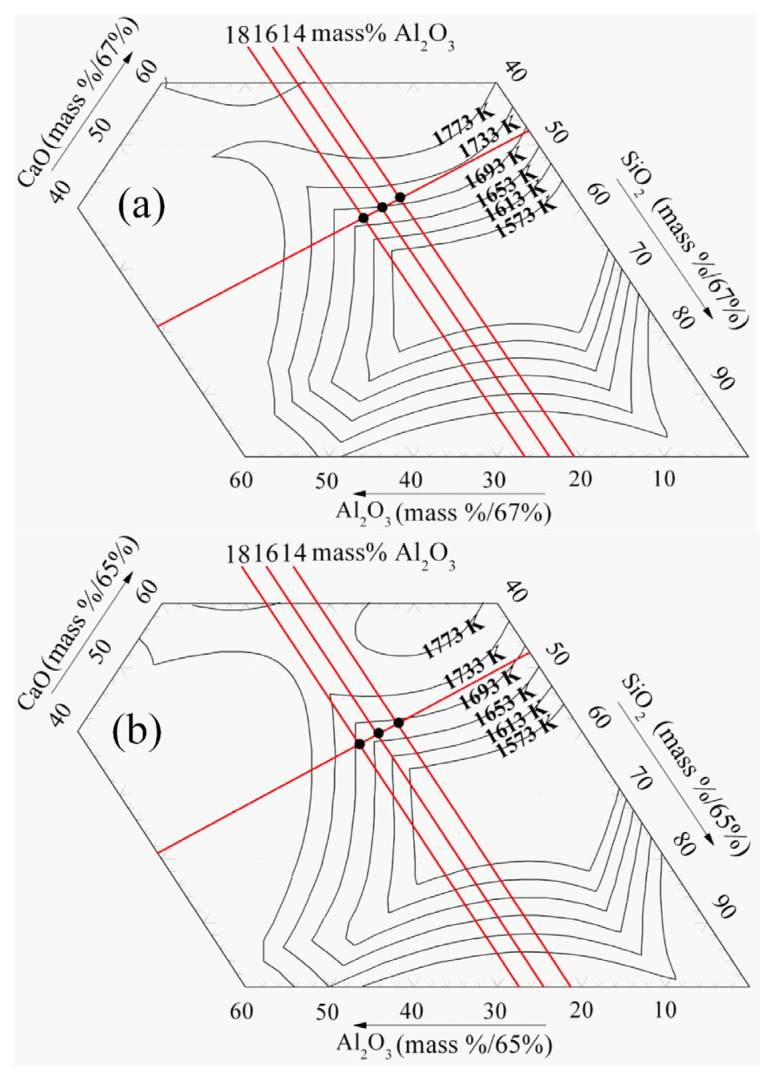
Phase diagram of CaO-SiO_2_-MgO-Al_2_O_3_-25 mass% TiO_2_ slag, K. (**a**) 8 mass% MgO; (**b**) 10 mass% MgO.

**Figure 9:**
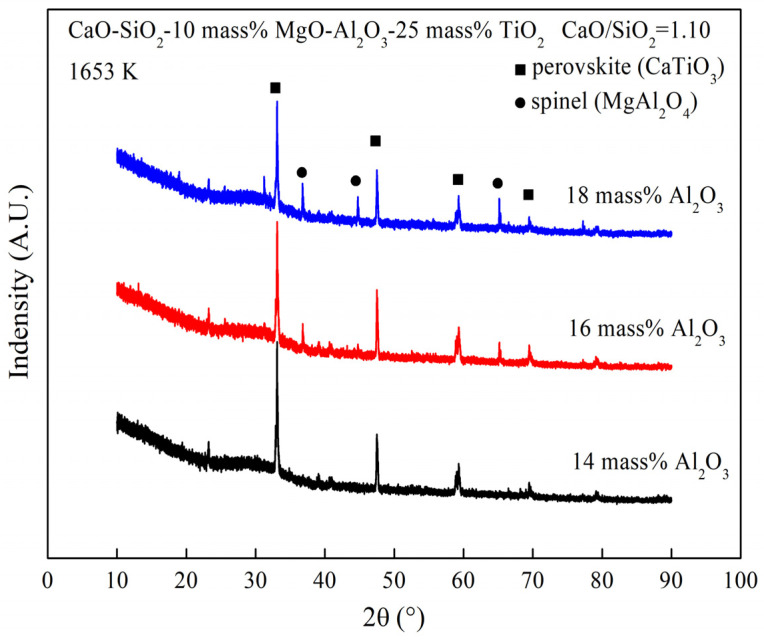
XRD curves of the slag containing different Al_2_O_3_ content at 1653 K.

The viscosity of the sample undergoes a significant increase when the temperature drops to 1653 K, which can be attributed to the solid phase appearing, as indicated in Figure 8 calculated by FactSage [23]. As illustrated in Figure 8a, at 1653 K, the liquidus temperature decreases with the increase in Al_2_O_3_ content, when the MgO content is 8 mass%. The reduction in solid phase content may be the primary reason for the viscosity decrease with the addition of Al_2_O_3_ content. When the Al_2_O_3_ content increases from 14 mass% to 18 mass% at 10 mass% MgO content, the primary crystal zone undergoes a change, as observed in Figure 8b. The XRD results depicted in Figure 9 show that the solid phase is converted from perovskite to perovskite and spinel when the Al_2_O_3_ content is added. In some instances, the intensity of the perovskite peak reduces with the introduction of the spinel. When the content of spinel in the slag is low, the effect of perovskite on viscosity is potentially greater than that of spinel. Hence, at 1653 K and 10 mass% MgO content, the addition of Al_2_O_3_ content induces a decrease in viscosity.

### 3.4. FT–IR Spectra of the Slag

**Figure 10:**
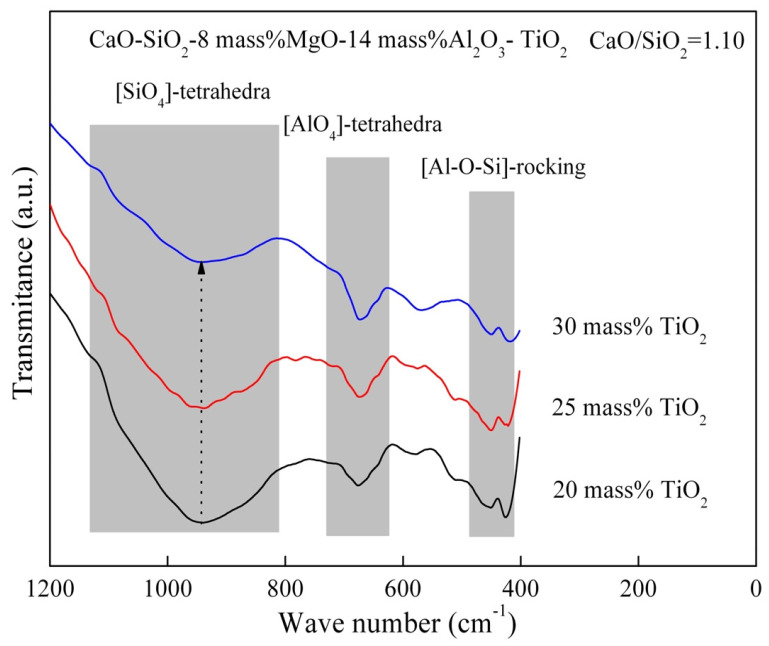
FT–IR transmittance spectra of the slag containing different TiO_2_ content at 1773 K.

In order to further understand the influence mechanism of the slag compositions on the viscosity, FT–IR spectra of the quenched slag at 1773 K were obtained. Figure 10 shows the FT–IR spectra of the slag containing different TiO_2_ content. The FT–IR spectrum of the slag can be divided into three crucial ranges according to the wave number. These ranges represent [SiO_4_]-tetrahedra between 1200 cm^−1^ and 800 cm^−1^, [AlO_4_]-tetrahedra between 730 cm^−1^ and 630 cm^−1^, and [Al-O-Si]-rocking between 480 cm^−1^ and 410 cm^−1^, respectively [29,30].

The depth of the FT–IR spectra between 1200 cm^−1^ and 800 cm^−1^ decreases with the increase in TiO_2_ content, which indicates that the network structure of [SiO_4_]-tetrahedra becomes simpler with TiO_2_ content addition. The depth of the peak representing the [Al-O-Si]-rocking is also decreased with the increase in TiO_2_ content. The effect of TiO_2_ content on [AlO_4_]-tetrahedra is insignificant. According to previous study, the existent forms of TiO_2_ in the slag include two types [12,31]. TiO_2_ as a basic oxide provides Ti^4+^ to depolymerize network structure or it as an acidic oxide forms simple network structure of TiO_4_^4−^. In the present study, TiO_2_ might be more inclined to existing as a network modifier and depolymerizes the silicate network structure. So, the viscosity decreases with TiO_2_ addition.

**Figure 11:**
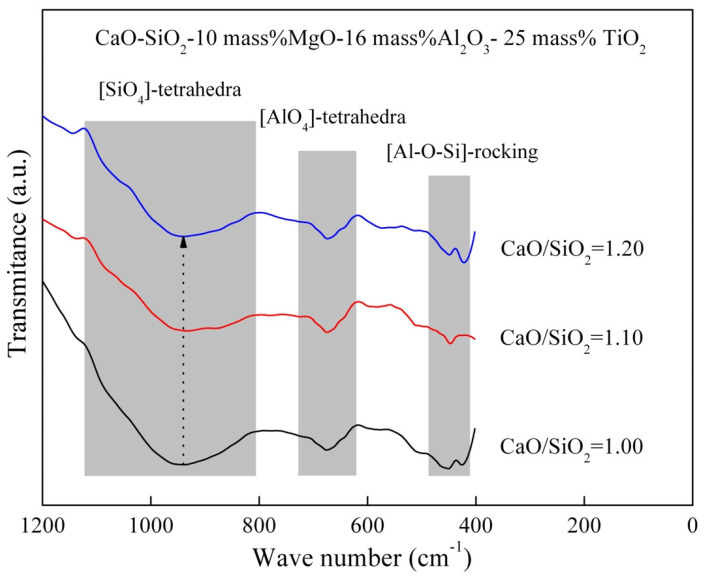
FT–IR transmittance spectra of the slag containing different basicity at 1773 K.

Figure 11 shows the FT–IR spectra of the slag with different basicity. The depth of the FT–IR spectra between 1200 cm^−1^ and 800 cm^−1^ decreases with the increase in basicity. Sometime, the depth of FT–IR spectra representing the [AlO_4_]-tetrahedra has hardly changed. It is similar to the effect of TiO_2_ on the network structure. The network structure of [SiO_4_]-tetrahedra should become simpler with the increase in basicity, which is consistent with the description in Figure 5.

**Figure 12:**
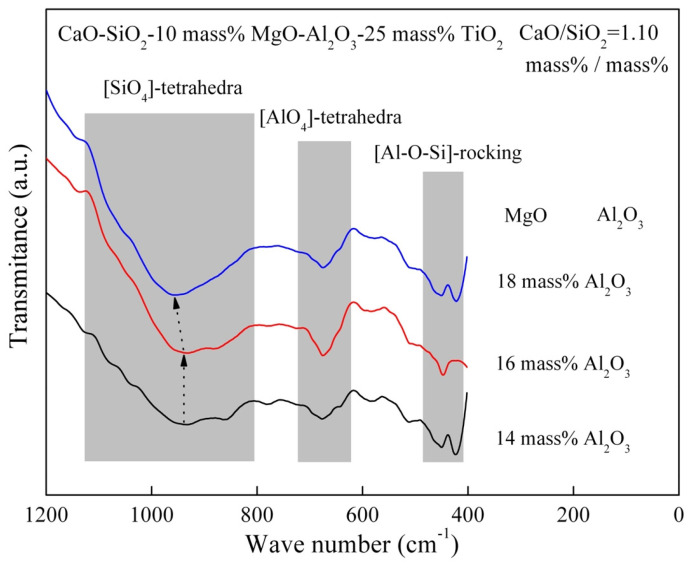
FT–IR transmittance spectra of the slag containing different Al_2_O_3_ content at 1773 K.

The FT–IR spectra of the slag with different Al_2_O_3_ contents are shown in Figure 12. The depth of the FT–IR spectra characterizing the [SiO_4_]-tetrahedra has hardly changed and it characterizing [AlO_4_]-tetrahedra significantly increases with the Al_2_O_3_ content from 14 mass% to 16 mass%. As Al_2_O_3_ content continues to 18 mass%, the depth of the FT–IR spectra characterizing the [SiO_4_]-tetrahedra does not only increase but also the central position of the peak of the FT–IR spectra has changed. Sometimes, the depth of the FT–IR spectra characterizing [AlO_4_]-tetrahedra decreases.

It is easily understandable that the network structure of [AlO_4_]-tetrahedra in the slag system increases as the Al_2_O_3_ content serves as an acidic oxide. However, when the Al_2_O_3_ content reaches 18 mass%, the network structure of [AlO_4_]-tetrahedra is reduced and the network structure of [SiO_4_]-tetrahedra becomes more complicated. This is because some of the Si^4+^ in [SiO_4_]-tetrahedra are replaced by Al^3+^, and Al^3+^ still exist in the form of [SiO_4_]-tetrahedra. As a result, a more intricate silicate network structure is formed while the network structure of [AlO_4_]-tetrahedra is reduced. It is evident that the effect of the silicate network structure on viscosity should be more pronounced than that of [AlO_4_]-tetrahedra. According to Figure 2 and Figure 12, the increase in viscosity with Al_2_O_3_ content from 14 mass% to 16 mass% is attributed to an increased proportion of [AlO_4_]-tetrahedra in the network structure, whereas the increase in viscosity with Al_2_O_3_ content from 16 mass% to 18 mass% is attributed to the more complex silicate network structure generated.

## 4. Conclusions

In this study, the viscosity of CaO-SiO_2_-MgO-Al_2_O_3_-TiO_2_ slag with different TiO_2_ content (10–30 mass%), Al_2_O_3_ content (14–16 mass%), and basicity (1.00–1.20) was measured. FactSage was used to draw the slag phase diagram and X-ray diffraction analysis was performed to understand the effect of calcium titanate and spinel solid phases on slag viscosity, and the relationship between network structure and viscosity was studied through FT–IR spectra.

(1) TiO_2_ might be more inclined to existing as a network modifier, and depolymerizes the silicate network structure in liquid slag. So, the viscosity decreases with the TiO_2_ content from 10 mass% to 30 mass% at high temperature.
(2) The viscosity of the slag increases as the content of Al_2_O_3_ increases from 14 mass% to 16 mass%. This is due to the formation of a network structure of [AlO_4_]-tetrahedra. However, when the Al_2_O_3_ content increases from 16 mass % to 18 mass %, there is a partial replacement of Si^4+^ in [SiO_4_]-tetrahedra with Al^3+^, while some Al^3+^ still exists in the form of [SiO_4_]-tetrahedra. As a result, the increase in viscosity can be attributed to the generation of a more complex silicate structure.
(3) The basicity of slag affects its viscosity, with higher basicity resulting in lower viscosity at temperatures above 1733 K. The decrease in viscosity is due to the reduction in size and number of silicate network structure caused by an increase in CaO content and a decrease in SiO_2_ content. The increase in solid phase content with increasing basicity results in an increase in viscosity at 1653 K, but when the amount of solid phase is small, it has almost no effect on the viscosity.
(4) At 1653 K, the solid phase in the current slag system includes perovskite and spinel, with perovskite playing an important role in viscosity.

## Figures and Tables

**Table 1:** The chemical composition of slags.

Sample Number	Chemical Compositions (Mass %)					R = m(CaO)/m(SiO_2_)
	CaO	SiO_2_	MgO	Al_2_O_3_	TiO_2_	
1	35.62	32.38	8.00	14.00	10.00	1.10
2	33.00	30.00	8.00	14.00	15.00	1.10
3	30.38	27.62	8.00	14.00	20.00	1.10
4	27.76	25.24	8.00	14.00	25.00	1.10
5	25.14	22.86	8.00	14.00	30.00	1.10
6	26.50	26.50	8.00	14.00	25.00	1.00
7	27.15	25.85	8.00	14.00	25.00	1.05
8	28.35	24.65	8.00	14.00	25.00	1.15
9	28.91	24.09	8.00	14.00	25.00	1.20
10	24.50	24.50	10.00	16.00	25.00	1.00
11	25.10	23.90	10.00	16.00	25.00	1.05
12	25.67	23.33	10.00	16.00	25.00	1.10
13	26.21	22.79	10.00	16.00	25.00	1.15
14	26.73	22.27	10.00	16.00	25.00	1.20
15	26.71	24.29	8.00	16.00	25.00	1.10
16	25.67	23.33	8.00	18.00	25.00	1.10
17	26.71	24.29	10.00	14.00	25.00	1.10
18	24.62	22.38	10.00	18.00	25.00	1.10

**Table 2:** Measured viscosity values of the CaO-SiO_2_-MgO-Al_2_O_3_-TiO_2_ slags.

Sample Number	Viscosity (Pa·s)			
	1653 K	1693 K	1733 K	1773 K
1	0.57	0.43	0.33	0.26
2	0.85	0.40	0.30	0.25
3	1.47	0.31	0.25	0.22
4	1.24	0.27	0.18	0.15
5	0.69	0.22	0.17	0.15
6	0.28	0.22	0.18	0.16
7	0.86	0.22	0.18	0.16
8	1.43	0.34	0.16	0.14
9	1.57	0.52	0.15	0.13
10	0.24	0.21	0.20	0.19
11	0.25	0.21	0.19	0.19
12	1.18	0.25	0.21	0.18
13	1.42	0.18	0.16	0.15
14	1.80	0.29	0.15	0.14
15	0.93	0.27	0.23	0.20
16	0.92	0.29	0.23	0.21
17	1.39	0.28	0.19	0.17
18	1.13	0.24	0.21	0.19

## Data Availability

The data presented in this study are available on request from the corresponding author.
